# Inhibition of p38 MAP kinase pathway induces apoptosis and prevents Epstein Barr virus reactivation in Raji cells exposed to lytic cycle inducing compounds

**DOI:** 10.1186/1476-4598-8-18

**Published:** 2009-03-09

**Authors:** Giulia Matusali, Giuseppe Arena, Alessandra De Leo, Livia Di Renzo, Elena Mattia

**Affiliations:** 1 Dept. of Public Health Sciences, University "La Sapienza", P. le A. Moro 5, 00185 Rome, Italy; 2 Dept. of Experimental Medicine and Pathology, University "La Sapienza", V. le Regina Elena 324, 00161 Rome, Italy

## Abstract

**Background:**

EBV lytic cycle activators, such as phorbol esters, anti-immunoglobulin, transforming growth factor β (TGFβ), sodium butyrate, induce apoptosis in EBV-negative but not in EBV-positive Burkitt's lymphoma (BL) cells. To investigate the molecular mechanisms allowing EBV-infected cells to be protected, we examined the expression of viral and cellular antiapoptotic proteins as well as the activation of signal transduction pathways in BL-derived Raji cells exposed to lytic cycle inducing agents.

**Results:**

Our data show that, following EBV activation, the latent membrane protein 1 (LMP1) and the cellular anti-apoptotic proteins MCL-1 and BCL-2 were quickly up-regulated and that Raji cells remained viable even when exposed simultaneously to P(BU)_2_, sodium butyrate and TGFβ. We report here that inhibition of p38 pathway, during EBV activation, led to a three fold increment of apoptosis and largely prevented lytic gene expression.

**Conclusion:**

These findings indicate that, during the switch from the latent to the lytic phase of EBV infection, p38 MAPK phosphorylation plays a key role both for protecting the host cells from apoptosis as well as for inducing viral reactivation. Because Raji cells are defective for late antigens expression, we hypothesize that the increment of LMP1 gene expression in the early phases of EBV lytic cycle might contribute to the survival of the EBV-positive cells.

## Background

Epstein Barr Virus (EBV), the causative agent of infectious mononucleosis, is associated with an increasing number of malignancies of epithelial and lymphoid origin that include Burkitt's lymphoma (BL), nasopharyngeal carcinoma, Hodgkin's lymphoma and immunoblastic lymphomas in posttransplant and AIDS patients [1]. Following primary infection, EBV infects epithelial cells where it undergoes lytic replication, and B cells, where it usually maintains a latent state [2]. All EBV-associated tumors have a predominantly latent pattern of viral gene expression. Three types of latent programs have been characterized depending on the differential expression of a limited set of viral genes. These include six nuclear antigens (EBNA1, 2, 3A, 3B, 3C and LP) and three membrane-associated proteins (LMP1, LMP2A and 2B) plus several small RNA species (EBERs).

*In vitro*, EBV infection of peripheral B lymphocytes results in their immortalization and continuous proliferation [3]. Among the latent proteins, LMP1 plays a prominent role in the process of EBV-associated oncogenesis. This integral membrane protein can cause transformation of rodent fibroblasts and epithelial cells *in vitro *[4,5] and induce development of B cell lymphoma or epidermal hyperplasia in transgenic mice [6,7]. By functioning as constitutively activated member of the tumor necrosis factor receptor (TNFR) family, through the cytoplasmic carboxy terminus LMP1 triggers several signaling pathways to alter cell growth and survival [8,9]. This viral oncoprotein stimulates NFkB, JNK, the JAK/STAT, PI3K/Akt, ERK1/2, and p38 mitogen activated protein kinase (MAPK) signal transduction cascades [10]; in addition, it regulates several downstream genes including anti-apoptotic genes such as bcl-2 [11,12], mcl-1 [13], A20 [14] and survivin [15].

Viral reactivation is initiated by the two immediate early proteins BZLF1 (ZEBRA or Zta) and BRLF1 (Rta) [16,17] that function as transcriptional activators of EBV early genes [18-20].

*In vitro*, latency can be disrupted by a variety of different agents such as phorbol esters, sodium butyrate, TGFβ, anti-immunoglobulins (anti-IgG) and calcium ionophores [21-23].

It has been reported that all these compounds induce apoptosis in EBV-negative cells but not in BZLF1-positive cells that appeared to be protected. Moreover, the antiapoptotic effect was prevented by treatment of the cells with inhibitors of viral DNA synthesis, leading to the hypothesis that a late EBV gene product might be responsible for survival of EBV-positive cells exposed to lytic cycle inducing compounds [24].

In this report we have further examined the connection between EBV lytic cycle induction and survival of the host cell aiming to detect viral gene products and/or signal transduction pathways involved in the protective effect. To focus on the early phases of EBV productive cycle, we used Burkitt lymphoma-derived Raji cells that, because of a deletion in EBV genome, support an abortive cycle, only allowing immediate early (IE) and early (E) genes expression [25]. We have previously shown that treatment of Raji cells with phorbol-12,13-dibutyrate (P(BU)_2_), sodium butyrate and TGFβ, activates EBV lytic cycle in more than 60% of the cell population [26]. We report here that following EBV activation, LMP1 and bcl-2 were promptly up-regulated and, despite the lack of viral late products, Raji cells were protected from apoptosis. We demonstrate that the suppression of p38 phosphorylation by its specific inhibitor caused a three fold increment of apoptosis. Furthermore, we found that inhibition of p38 signaling pathway largely prevented EBV lytic gene expression. These findings indicate that p38 MAPK plays a key role both in EBV activation as well as in host cell survival. In addition, the increment of LMP1 expression at the onset of the lytic phase suggests that the latent viral oncogene might contribute to preserve cell viability in the early stages of EBV productive cycle.

## Results

### Activators of EBV lytic cycle induce apoptosis in EBV-negative Burkitt's lymphoma cells

EBV lytic cycle can be induced in Burkitt's lymphoma cells by treatment with phorbol esthers, anti-immunoglobulin, sodium butyrrate or TGFβ. Although each of these agents cause apoptosis, it has been reported that lytic EBV gene expression prevents the cells to undergo the programmed cell death [24].

**Figure 1:**
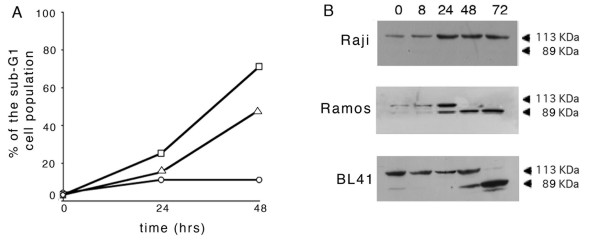
**Evaluation of apoptosis in BL cells during exposure to EBV lytic cycle inducing compounds**. EBV-positive Raji (circles) and EBV-negative Ramos (squares) and BL41 (triangles) cells were treated with P(BU)_2_, sodium butyrate and TGF-β as described in the Methods. At the indicated times (hours), samples were collected and analyzed for (A) cytofluorimetric determinations of sub-G1 events after PI staining or (B) immunodetection of native (113 KDa) and apoptotic (89 KDa) forms of PARP by Western blot. The results are representative of those obtained in at least three independent experiments.

To further investigate the protective effect exerted by EBV infection, we treated EBV-positive Raji and EBV-negative Ramos and BL41cells with P(BU)_2_, sodium butyrate and TGFβ, a combination of agents able to trigger, in the former, EBV immediate early (IE) and early antigen (EA) expression in more than 60% of the population [26]. In the cells exposed to these compounds, we monitored apoptosis by analyzing in a cytofluorimeter the sub-G1 events and by Western blotting the proteolytic cleavage of poly(ADP-ribose) polymerase (PARP). Fig. 1A illustrates the results of a representative experiment where the percentages of cells with a sub-G1 DNA content were measured in the samples collected after 24 and 48 hours of incubation with EBV lytic cycle inducing agents. It appears that the fraction of EBV-negative cells with an hypodiploid DNA content increased with the incubation time. In particular, after 48 hour, about 50% of BL41 and 70% of Ramos cells were found in the sub-G1 peak. In contrast, treatment of EBV-positive Raji cells with P(BU)_2_, sodium butyrate and TGFβ for the same lengths of time did not significantly affect the percentage of the sub-G1 population that remained at about 10% for the entire time of treatment. Results similar to those shown in Fig. 1A were obtained when apoptosis was detected by annexin V-FITC binding to phosphatidylserine (data not shown).

In addition, Western blotting analysis of PARP cleavage carried out on the lysates of cells treated as mentioned above, is illustrated in the image of Fig. 1B. It appears that the 89 KDa apoptotic form of the protein was generated in EBV negative Ramos and BL41 cells after incubation with the lytic cycle activators for 24 and 48 hours, respectively. Conversely, only the full form of PARP was observed in Raji cells even when exposed to P(BU)_2_, sodium butyrate and TGFβ for up to 72 hours.

In order to confirm that the different apoptotic response in Raji cells was related to EBV infection, we compared apoptosis in EBV-positive and EBV-negative Akata cells, after treatment with anti-IgG. Table 1 reports the results of a representative experiment as percentages of apoptotic cells (annexin V-positive) and apoptotic plus necrotic (annexin V and propidiun iodide positive) cells measured by flowcitometry at 0, 24 and 48 hours. It is evident that at 24 hours, the fraction of apoptotic cells in the EBV-infected population was about one fifth of that measured in the absence of the virus, indicating that EBV protects Akata cells from IgG cross-linking induced apoptosis. This result was confirmed by the analysis of the values measured at 48 hours indicating that post-apoptotic processes in the EBV-negative cellls resulted in a much larger increment of cells permeable to propidium iodide. Results similar to those shown in the Table were obtained when EBV lytic cycle induction was triggered by P(BU)_2_, sodium butyrate and TGFβ.

**Table 1:** Evaluation of apoptosis in EBV-positive and EBV-negative Akata cells treated with anti-IgG

	T ^a^	Ann V ^b^	Ann V + PI ^c^
Akata	0	4	5
EBV	24	10	12
positive	48	22	25

Akata	0	5	16
EBV	24	54	26
negative	48	8	75

^a ^Time of exposure of the cells to anti-IgG

^b ^AnnexinV-positive cells (%)

^c ^Annexin V plus propidium iodide-positive cells (%)

### Expression of viral and cellular antiapoptotic genes during EBV lytic cycle induction

**Figure 2:**
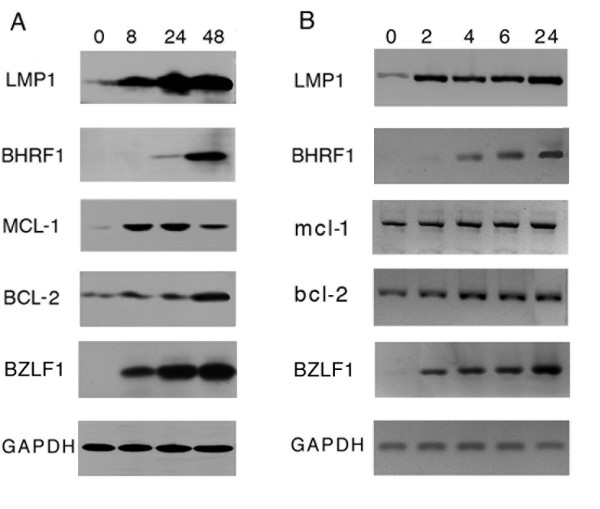
**Expression of viral and cellular antiapoptotic genes during EBV lytic cycle activation**. (A) Raji cells were exposed to lytic cycle inducing agents as described in the Methods. At the indicated times (hours), (A) cell lysates were resolved by 10% acrylamide SDS-PAGE, blotted on membrane and probed with specific antibodies; (B) purified RNA was reverse transcribed and cDNA amplified with the specific primers (see Methods).

Because EBV-positive Raji cells appeared to be protected from apoptosis despite incubation with multiple EBV lytic cycle inducing compounds, we next examined the levels of expression of both viral as well as cellular antiapoptotic genes. Among the viral genes, LMP1, BHRF1 and BALF1 have been reported to suppress apoptosis. However, BALF1 is not expressed in Raji cells because comprised in the genome deletion that accounts for the replication defect in this strain [25]. Therefore, we analyzed LMP1 and BHRF1 expression in Raji cells collected at different times after addition to the culture of EBV lytic cycle activators. Fig. 2A shows that the immediate early antigen BZLF1 (34 KDa) is rapidly expressed after EBV induction and high levels of the protein are detected in the samples collected after 8 hours of incubation with the inducers. Similarly, the levels of LMP1 protein (63 KDa) dramatically increased after 8 hours of exposure of the cells to P(BU)_2_, sodium butyrate and TGFβ and raised further, reaching at 48 hours values about 10 times higher than those measured in latently-infected cells (time 0). Conversely, BHRF1 (17 KDa) was detectable only 24 hours after the addition of the lytic cycle activators and its level increased between 24 and 48 hours.

Because LMP1 induces the cellular anti-apoptotic genes mcl-1 and bcl-2 [13], we examined their expression during EBV lytic cycle activation. The levels of MCL-1 (43 KDa) largely increased in the samples collected at 8 and 24 hours but started to decline thereafter, confirming the previously reported transient increment following LMP1 expression. In contrast, BCL-2 levels slightly raised during the first 24 hours of incubation of the cells with the inducers, but in the samples collected at 48 hours, the intensity of the specific band (29 KDa) was about three times higher than that measured in the untreated cells (time 0).

To further investigate the time-course and the regulation of the expression of the viral and the cellular anti-apoptotic genes, we measured mRNA levels by RT-PCR to assess whether the up-regulation of LMP1, MCL-1 and BCL-2 proteins was related to increased transcription of the corresponding genes. As it appears from Fig. 2B, a semi-quantitative evaluation of the transcripts indicates that besides EBV lytic genes, also LMP1, Mcl-1 and Bcl-2 expression is regulated at the transcriptional level. However, while LMP1 mRNA levels increased concomitantly with BZLF1 gene expression after 2 hours exposure of the cells to EBV lytic cycle activators, BHRF1 transcripts appeared only after 4 hours. Similar times of incubation of the cells with the inducing agents were necessary to detect a slight but consistent increment of mcl-1 and bcl-2 mRNAs.

### EBV activation and ROS production

**Figure 3:**
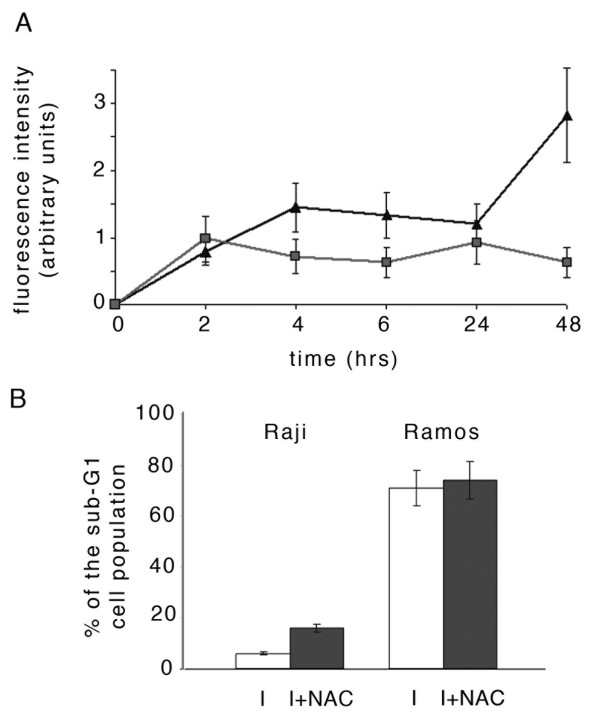
**ROS production in Raji and Ramos cells exposed to EBV lytic cycle activators**. (A) Cells were treated with P(BU)_2_, sodium butyrate and TGFβ. At the indicated times, samples of Raji (triangles) and Ramos (squares) were incubated with an oxidation-sensitive probe and the oxidized fluorescent derivative analyzed by flowcytometry; (B) cells exposed to EBV lytic cycle activators in the absence (open bars) or in the presence (filled bars) of N-acetyl cysteine for 48 hours were fixed and PI stained for cytofluorymetric determination of sub-G1 events. Error bars are the means ± SD of three independent experiments.

Variations in the redox state play an important role in modulating cell survival and apoptosis. Therefore, we asked whether the different percentage of apoptotic cells measured in Raji and Ramos exposed to EBV activating compounds, was related to variations in the levels of reactive oxygen species (ROS). To this end, Raji and Ramos cells were incubated with P(BU)_2_, sodium butyrate and TGFβ for up to 48 hours. Within this period of time, cell samples were collected, subjected to an oxidation-sensitive probe and analyzed by flowcitometry. Fig. 3A shows that in Raji cells ROS production increased in the first four hours, reached a plateau and augmented to a greater extent between 24 and 48 hours. In contrast, in Ramos cells ROS levels were lower, both at early as well as at late times after the addition to the culture of the inducing agents.

In particular, at 48 hours, the values measured in the EBV negative cell line were about one third of those measured in Raji cells. To further evaluate these results, we analyzed the sub-G1 population in Raji and Ramos cells exposed to the inducers for 48 hours, in the absence or in the presence of N-acetyl cysteine. The bargraph of Fig. 3B indicates that a slightly higher percentage of apoptosis was measured in Raji cells when EBV lytic cycle was induced in the presence of the antioxidant molecule. However, N-acetyl cysteine was not able to reduce apoptosis in Ramos cells, clearly confirming that cell death of EBV-negative cells was not related to higher production of ROS.

### Contribution of signal transduction pathways to protection of induced Raji cells from apoptosis

**Figure 4:**
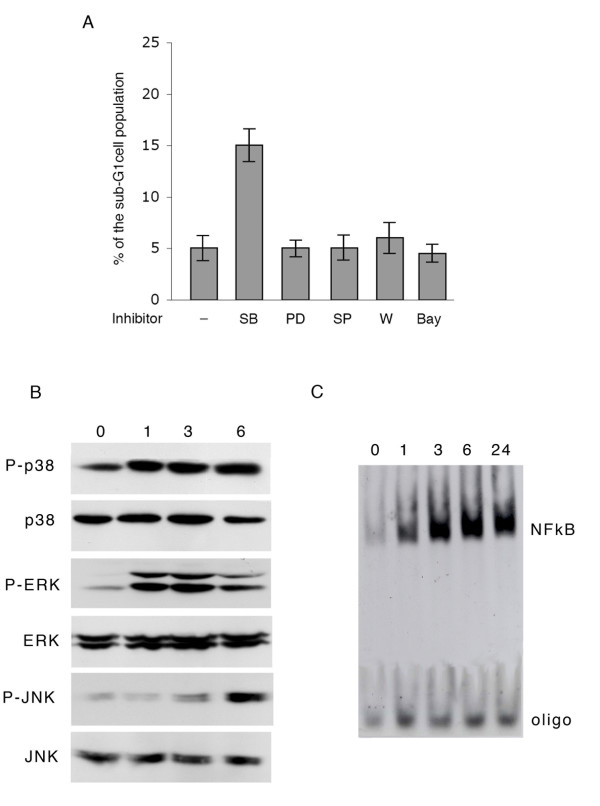
**Contribution of signal transduction pathways to cell viability during exposure of Raji cells to EBV lytic cycle activators**. (A) Cells were pretreated for 15 min with the specific inhibitors of p38 (SB), ERK (PD), JNK (SP), PI3K (W) or NFkB (Bay) and then exposed to P(BU)_2_, sodium butyrate and TGFβ as described in the Methods. After 48 hours, cells were fixed and PI stained for cytofluorymetric determination of sub-G1 events; (B) cells were incubated with P(BU)_2_, sodium butyrate and TGFβ. At the indicated times (hours), cell lysates were prepared and the phosphorylation pattern of p38, ERK and JNK evaluated by Western blot analysis with the specific antibodies for each protein and its phosphorylated (P-) form; (C) cells were treated as in B. At the indicated times, cell extracts were incubated with a consensus NFkB binding site oligonucleotide (oligo) to evaluate NFkB activity by EMSA (see Methods).

In order to assess the role played by different signal transduction pathways in protecting induced Raji cells from apoptosis, EBV induction was carried out during exposure of the cells to specific inhibitors of the main signaling molecules known to be activated during the latent and/or the early phases of the lytic EBV infection. Therefore, Raji cells were incubated with P(BU)_2_, sodium butyrate and TGFβ for 48 hours in the presence of SB203580, PD98059, SP600125 or Wortmanin, specific inhibitors of p38 MAPK, ERK, JNK and PI3K, respectively or with Bay 117082, inhibitor of NFkB pathway. Thereafter, cells were subjected to cell cycle analysis and the apoptosis measured as the sub-G1 fraction of the cell population. As it appears from Fig. 4A, after exposure for 48 hours to EBV lytic cycle activators about 5% of Raji cells was characterized by a hypodiploid DNA content. This percentage did not vary significantly when EBV induction was carried out in the presence of agents that inhibit ERK, JNK, PI3K and NFkB pathways. In contrast, the addition of SB203580 at a concentration proved not to be toxic on latently-infected cells, causes a three fold increment of apoptosis, indicating that p38MAPK pathway highly contributed to protect EBV-positive Raji cells from apoptosis. To assess the specificity of p38 signaling cascade in the protective effect on Raji cells, we verified the activation of the different pathways by analyzing the phosphorylation pattern of p38, ERK and JNK, as well as NFkB activity, at different times after exposure of the cells to the inducing agents. As shown in Fig. 4B, the results of Western blots carried out with specific antibodies for p38, ERK, JNK and for the phosphorylated forms of the three MAPKs, indicate that all of them were activated. In particular, phosphorylation of both p38MAPK and ERK strongly increased at 1 hour after induction of EBV lytic cycle; however, the levels of phospho-p38 remained high up to six hours, while phospho-ERK increment appeared to be transient. Moreover, JNK activation occurred at later times (3 hours), but increased thereafter. Experiments carried out to verify the efficacy of p38 inhibitor showed that at the concentration used, SB203580 largely suppressed p38 phosphorilation without affecting JNK or ERK activation (data not shown). In addition, NFkB-DNA binding activity analyzed by EMSA (Fig. 4C) revealed an increment in the signal corresponding to the specific complex indicating that induction of EBV lytic cycle led to prompt and persistent activation of the transcription factor NFkB.

### Inhibition of signal transduction pathways and EBV lytic cycle activation

Because p38 signaling cascade appeared to be involved in the protection of EBV-positive cells from apoptosis when exposed to lytic cycle inducing compounds, we asked whether inhibition of this pathway could affect EBV lytic cycle activation. To assess this point, after a 48 hours incubation of Raji cells with P(BU)_2_, sodium butyrate and TGFβ in the presence of either SB203580, PD98059, SP600125, Wortmanin, or Bay 117082, cell samples were fixed on slides and treated for immunofluorescence staining with antibodies recognizing EBV early antigens.

**Figure 5:**
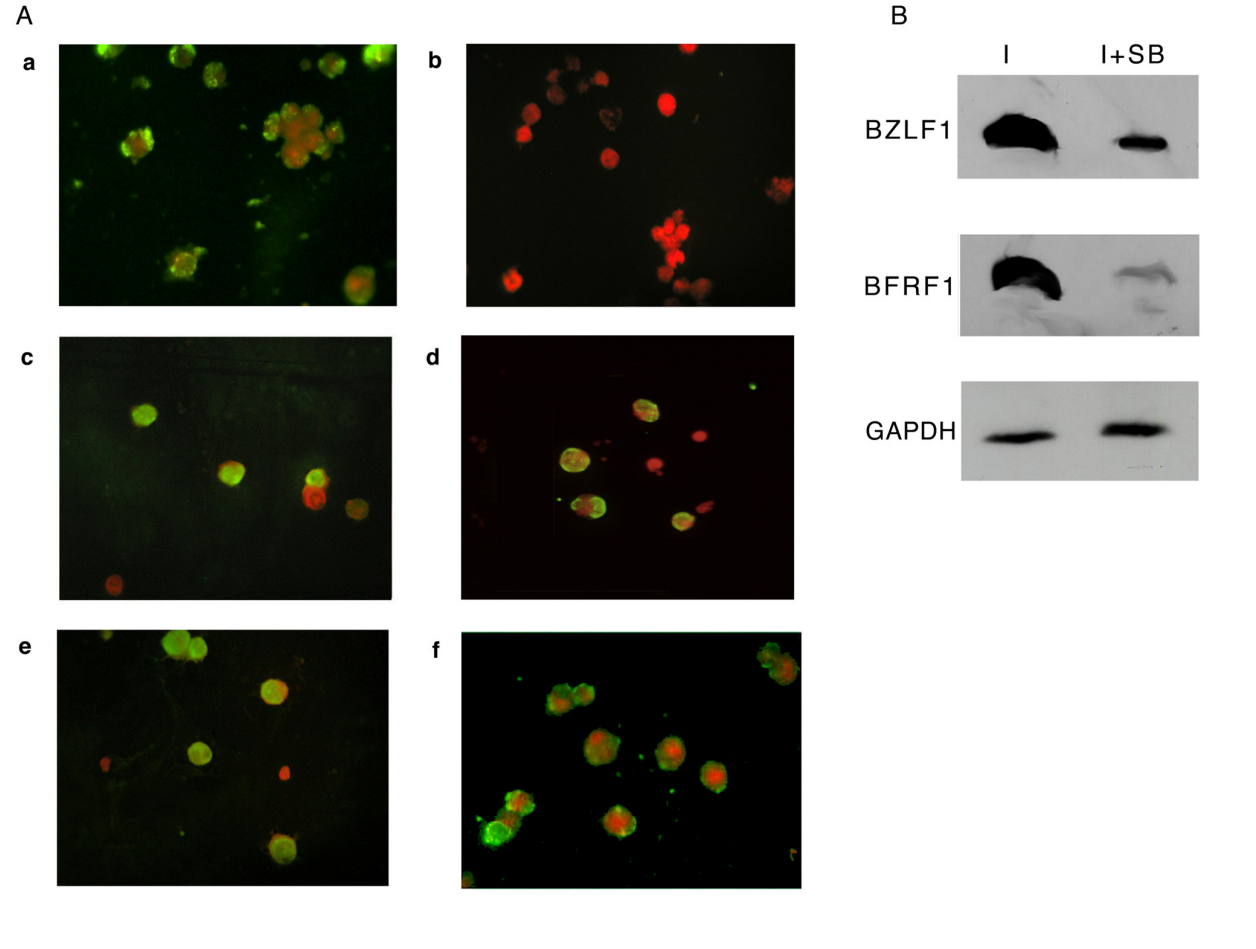
**EBV activation in the presence of inhibitors of signal transduction pathways**. Raji cells were treated as described in Fig. 4 A. (A) Fixed cells were stained for immunofluorescence analysis with FITC-labelled antibodies specific for EBV early antigens: a) no inhibitor; b), c), d), e), f) plus inhibitor of p38, ERK, JNK, PI3K or NFkB, respectively; (B) Raji cells were exposed to EBV activators in the absence (I) or in the presence (I+SB) of p38 inhibitor. At 48 hours, cell extracts were resolved onto a 10% acrylamide gel by SDS-PAGE, blotted and probed with BZLF1 or BFRF1 antibodies. Hybridization with GAPDH antibodies was used as loading control. Specific signal were visualized by ECL.

In the panel of Fig. 5A, the results of a representative experiment are shown. The image in a) shows that after 48 hours incubation with lytic cycle inducers, FITC-labeled antibodies detected EA in a large percentage of the cell population as previously reported [26]. EA expression was also detected when lytic cycle induction was carried out in the presence of inhibitors of ERK, JNK, PI3K or NFkB pathway (pictures c, d, e and f).

In contrast, FITC fluorescence signal was absent in the samples of cells incubated with SB203580 (picture b), indicating that activation of p38 pathway is essential for induction of EBV lytic cycle. To further analyze this result, we measured the expression of BZLF1 and BFRF1, an immediate early and an early antigen, respectively, of EBV lytic program. As it appears from Fig. 5B, after a 48 hours treatment with the inducers in the presence of SB203580, the levels of both BZLF1 and BFRF1 were dramatically reduced as compared to those measured in the absence of the p38 inhibitor.

## Discussion

In this study we have reexamined the relationship between cell survival and EBV lytic cycle to determine viral factors and/or signal transduction pathways involved in the protection of EBV-infected cells from apoptosis during viral reactivation. We attempted to investigate this issue in Raji cells that, treated with multiple agents, support a massive induction of an abortive EBV lytic cycle [25,26] characterized by the expression only of the viral IE and E genes. We show here that in contrast to EBV-negative BL cell lines, EBV-positive Raji and Akata cells do not die, even after prolonged exposure to the activating agents. These results confirm and strengthen what previously observed; in addition, because late antigens are not expressed in Raji cells, our data exclude the possibility that a late viral product is involved in the protective effect.

ROS have been implicated in the regulation of several cellular functions, including intracellular signaling, transcription activation, proliferation, and apoptosis [27-29]. For what concerns apoptosis, ROS can act as a reactive signal, denature DNA and alter intracellular organelles [30-32].

We asked whether treatment of the cells with EBV lytic cycle activators would differently affect ROS levels in EBV-positive and negative BL cells to account for survival or apoptosis. Recently, it has been reported that *in vitro *EBV infection is associated with ROS production [33]; furthermore, EBV-positive BL have been shown to express high levels of MAPK and ROS while EBV-negative BLs exhibit activation of PI3-kinase, but do not have elevated levels of ROS [34]. Our data show that upon exposure to lytic cycle inducing compounds, ROS production remains higher in the EBV-positive as compared with the EBV-negative cells; in addition, pretreatment with N-acetyl cystein (a ROS inhibitor) did not block cell death of EBV-negative BL cells, further indicating that apoptosis occurring in these cells is not related to ROS production.

We next looked for viral factors and signal transduction pathways that could play a role in the protection of EBV-positive cells from apoptosis upon induction of EBV lytic cycle.

Many gamma herpesviruses appear to express their BCL-2 homologues early in the lytic replication cycle, raising the possibility that herpesvirus BCL-2 proteins prolong cell survival to allow production of greater numbers of progeny. Here we show that EBV BCL-2 homologue BHRF1 is expressed only 24 hours after addition of lytic cycle activators, while a dramatic increment of LMP1 is detectable as soon as after 8 hours. Moreover, the transcriptional activation of LMP1 in the lytic cycle results in the up-regulation of the MCL-1 and BCL-2 cellular anti-apoptotic proteins as previously described to occur in LMP1-transfected B lymphocytes [13].

The increment of LMP1 in the early phases of EBV lytic cycle has been previously reported [35,36]. However, in contrast to the knowledge of the roles during EBV latency, little is known about the biological significance of LMP1 up-regulation during the lytic cycle of viral replication. It has been reported that loss of LMP1 severely impairs the release of viral particles [37]. Other studies have shown that LMP1 expression inhibits EBV lytic cycle induction and progress [38]. Furthermore, a number of reports indicate that LMP1 expression levels directly affect the ability to stimulate signaling and cell proliferation positively, or to inhibit protein synthesis and induce cytostasis [39,40].

Far from being completely elucidated, it is conceivable that LMP1 up-regulation during EBV lytic cycle induction serves to prepare a suitable cell environment for virus replication by promoting cell survival or by triggering some essential signaling pathways. In this respect, our preliminary results obtained in cells exposed to EBV lytic cycle activators in the presence of LMP1-siRNA indicate that the latter is able to prevent LMP1 up-regulation and increase the percentage of apoptotic cells. Moreover, immunofluorescence experiments carried out on Raji cells exposed to EBV lytic cycle activators confirmed that BZLF1 and high levels of LMP1 are co-expressed in the same cell (data not shown).

We show here that the inhibition of p38 MAPK at the onset of EBV lytic cycle, increases by three fold the percentage of apoptotic Raji cells indicating that p38 phosphorylation largely contributes to protect EBV-positive BL cells during the early phases of EBV activation. It is well known that p38 MAPK as well as NFkB signaling transduction cascades are triggered by LMP1 to inhibit apoptosis and promote cell survival [10,41]. Our data indicate an increment of NFkB activity upon induction of EBV lytic phase of infection. However, evidence has been provided showing that treatment of Kaposi's sarcoma-associated herpesvirus (KSHV) or EBV latently-infected lymphocytes with an NFkB inhibitor leads to virus reactivation, suggesting that high NFkB activity levels contribute to maintenance of the viral latency state [42]. It can be hypothesized that the apparent discrepancy between our results and what was previously reported strictly depends on the cellular context. In this respect, treatment of Raji cells with an NFkB inhibitor did not lead to EBV reactivation in Raji cells (unpublished observations).

We also report that inhibition of p38 prevents EBV lytic cycle induction in Raji cells indicating the prominent role that this signaling pathway plays in the process of EBV reactivation. Data have been provided demonstrating that siRNA targeting p38MAPK blocks p38 phosphorylation and BZLF1 expression induced by TPA in EBV-positive epithelial cells [43]. Furthermore p38 inhibition prevented efficient disruption of viral latency by surface IgG crosslinking in Akata cells [44]. Our results, obtained in Raji cells in which the virus is activated with three lytic cycle inducing agents, confirm and extend the essential involvement of p38 activation in the break of viral latency. However, because Raji cells are exposed to multiple agents, each one able to trigger BZLF1 expression, it is likely the effector being downstream of BZLF1 activation.

The immediate early genes BZLF1 and BRLF1 activate one another's transcription and together trans-activate EBV early genes. They both induce phosphorylation of p38 kinase as well as JNK to activate the cellular transcription factor ATF2 [44]. It has also been found that, in contrast to BZLF1, the ability of BRLF1 expression vector to induce lytic EBV infection is markedly reduced by inhibition of either p38 or JNK pathways [44]. From our data it seems conceivable that p38 inhibitor might prevent EBV activation by interfering with p 38-mediated transcription of BZLF1 by BRLF1. However, the delayed phosphorylation of JNK with respect to the other MAPKs and the evidence that EBV activation occurs also in the presence of JNK inhibitor, indicate that this signal transduction pathway is not involved in the early phases of lytic infection in Raji cells.

## Conclusion

In conclusion, we report here that at the onset of EBV lytic cycle, p38 MAPK activation plays an important role both for protecting EBV-infected Raji cells from apoptosis, as well as for promoting EBV lytic gene expression. Our data strongly suggest that the increment of LMP1 protein occurring upon EBV reactivation may contribute in a relevant way to both events. Studies are currently carried out to test this hypothesis by elucidating the function of LMP1 up-regulation in the switch from latency to EBV lytic program.

## Methods

### Cell culture and treatment with EBV lytic cycle activators

EBV-positive Raji and Akata and EBV-negative Ramos, BL41 and Akata, are Burkitt's lymphoma (BL)-derived cell lines. They were cultured in RPMI 1640 medium containing 5% fetal calf serum (FCS) and antibiotics, in a 5% CO2 atmosphere and maintained at a cell density of 3.5 × 10^5^/ml.

Cells, at a density of 5 × 10^5 ^cells/ml, were incubated in RPMI 1640/2,5% FCS with P(BU)_2 _(Sigma), sodium butyrate (Sigma) and TGF-β2 (Genzyme, sp.act. 5 × 10^7 ^U/mg) at the final concentrations of 20 ng/ml, 2 mM and 0.04 ng/ml, respectively.

In addition, EBV-positive and EBV-negative Akata cells at a density of 2 × 10^6^/ml were incubated for 2 hours with 100 μg/ml of anti human IgG (Sigma) and subsequently diluted to 10^6 ^cells/ml. At different times, cell samples were collected and analyzed as described below. Cell viability was assessed by trypan blue exclusion.

### Cytofluorimetric analysis

The percentage of non viable cells was determined by FACS analysis after DNA staining with propidium iodide (PI). Cell samples were washed with phosphate buffer saline (PBS) and centrifuged for 5 min at 300 × g. The cell pellet was fixed for 1 h at 4°C with 70% ethanol, washed with PBS, and treated for 1 hour with 100 μg/ml of PI and 100 μg/ml RNase. DNA content was assessed by using an Epics Coulter XL flowcytometer. Apoptotic cells (pre-G1 peak) were considered as showing a DNA content less than 2N. Alternatively, early apoptosis was evaluated by annexin V-FITC apoptosis detection kit (Sigma) that measures annexin V binding to phosphatidylserine in conjunction with propidium iodide staining, according to the accompaining procedure.

### Western blot analysis

Cells (about 10^6^) were collected and washed with PBS before being lysed in Laemmli buffer. To detect phosphorylated proteins, cells were resuspended in lysis buffer (0,1% NaN_3_, 1 mM CaCl_2_, 1 mM MgCl_2_, 150 mM NaCl, 0.5 μM aprotinin, 4 μM leupeptin, 2 mM PMSF, 10 mM NaF, 10 mM iodoacetamide, 1 mM Na_3_VO_4_, 1% Triton-X 100 in PBS) and kept for 30 min on ice. Equal amounts of proteins (70 μg), as determined by a modified Lowry assay (RC DC protein assay, BioRad), were resolved on a 10% acrylamide gel by SDS-PAGE and transferred to PVDF membrane. The blots were incubated with PBS/0.1% Tween 20/5% non fat milk for 1 hour before being probed in the same solution with the following primary antibodies: anti LMP1 (1: 7500, PD Pharmigen), anti BCL-2 (1: 200, Dako), anti PARP (1: 5000, Alexis), anti MCL-1 (1: 200, Santa Cruz), anti BHRF1 (1: 20, kind gift from Dr. Kremmer, GSF-Forschungszentrum, Munich, Germany), anti p38 (1: 500, Santa Cruz), anti P-p38 (1: 500, Cell Signaling), anti ERK and anti P-ERK (both 1: 1000, Cell Signaling), anti JNK (1:1 000, Santa Cruz), anti P-JNK (1: 500, Santa Cruz), anti BZLF1 (1: 100, Argene), or anti BFRF1 (1: 1000, kindly provided by Dr. A. Farina, Dept. of Experimental Medicine, Univ. of Rome "Sapienza", Italy), for 1 hour at 25°C. The blots were further incubated for one hour with horseradish peroxidase conjugated anti-mouse or anti-rabbit (both 1: 5000, Amersham) or anti rat (1: 10000, Jackson IR). The specific signals were visualized by ECL detection kit (Amersham).

### RT-PCR experiments

Total RNA was isolated from Raji cells as previously described [45]. The specific primers for mcl-1, bcl-2, LMP1 and GAPDH, the PCR conditions and the size of the amplified sequences have been previously reported [45]. PCR products were loaded onto a 1.5% agarose gels containing 0.5 μg/ml ethidium bromide and visualized under UV light.

### Production of Reactive Oxigen Species (ROS)

Aliquots (2.5 × 10^5^) of untreated cells or cells incubated with EBV lytic cycle inducing compounds in the absence or in the presence of 2.5 mM N-acetyl cysteine, were collected and washed three times in 5 mM HBSS buffer pH 7.4 before being incubated with 1 μM 5,6-carbossy-2',7'-diclorohydro fluoresceine diacetate (DCFH-DA, Molecular Probes, Eugene, OR, USA) for 15' at room temperature. ROS production was analyzed by flowcytometry (EPICS Coulter-XL, FL, USA, excitation: 488 nm, emission: 530 nm) by measuring the oxidized fluorescent derivative DCF [46]. The levels of ROS detected in untreated cells or in H_2_O_2 _treated cells have been considered as basal level and positive control, respectively, to determine ROS production following EBV lytic cycle induction.

### Inhibition of signal transduction pathways

Samples of Raji and Ramos cells at a density of 1 × 10^6^/ml, were incubated for 15' at 37°C, in the presence of 10 μM SB203580 (SIGMA), 10 μM PD98059, 10 μM SP600125 or 1 μM Wortmanin (all from Calbiochem), specific inhibitors of p38 MAPK, ERK, JNK and PI3K, respectively, or with 5 μM Bay 117082 (Sigma), inhibitor of NFkB. Cells were then diluted with an equal volume of RPMI 1640 medium containing EBV lytic cycle inducing agents. After 48 hours, cell samples were collected and analyzed by Western blot, flowcytometry and fluorescence microscopy.

### Electrophoretic Mobility Shift Assay (EMSA)

Whole cell extracts were obtained after lysis in a high salt extraction buffer (50 mM Tris-HCl pH 7.5, 400 mM NaCl, 1 mM EDTA, 1 mM EGTA, 1% Triton X100, 0.5% NP40, 10% glycerol, 2 mM DTT, 2 μM aprotinin, 2 μM leupeptin, 1 mM Na_3_VO_4_, 2 μM pepstatin, 1 mM PMSF). 20 μg were incubated with 30 fmol of a DIG-labeled (DIG oligonucleotide 3' end-labeling kit, Roche Applied Science) kB DNA probe [47], in a binding buffer (20 mM Tris-HCl pH 7.5, 2 mM EDTA, 10% glycerol) containing 1 μg BSA, 0.5 μg poly d(I-C), for 20 min at room temperature. Complexes were resolved by nondenaturing 4% polyacrylamide gel electrophoresis, transferred to nylon membrane and detected by chemiluminescence (DIG luminescent detection kit, Roche Applied Science).

### Fluorescence microscopy

Treated cells were smeared on slide, fixed and permeabilized with methanol:acetone (2:1) for 5 min at -20°C and then stained with FITC-conjugated F6-Ester 2 antibodies recognizing EBV early antigens (EA), as previously described [48]. Slides were mounted with 50% glycerol in PBS and analyzed with a Leica DM4000 fluorescence microscope equipped with a FX 340 digital camera.

## Competing interests

The authors declare that they have no competing interests.

## Authors' contributions

GM carried out most of the experimental work and contributed to draft the manuscript. GA and ADL equally contributed to the present work by accomplishing cell culture treatments and helping to elaboration of data. LDR carried out cytofluorimetric analysis. EM participated and coordinated the study, compiled and finalized the manuscript. All authors read and approved the final manuscript.
